# Paediatric Home Artificial Nutrition in Italy: Report from 2016 Survey on Behalf of Artificial Nutrition Network of Italian Society for Gastroenterology, Hepatology and Nutrition (SIGENP)

**DOI:** 10.3390/nu10091311

**Published:** 2018-09-16

**Authors:** Antonella Lezo, Teresa Capriati, Maria Immacolata Spagnuolo, Laura Lacitignola, Irina Goreva, Grazia Di Leo, Nicola Cecchi, Paolo Gandullia, Sergio Amarri, Maria Luisa Forchielli, Valeria Dipasquale, Barbara Parma, Simona Gatti, Elisa Ravaioli, Silvia Salvatore, Martina Mainetti, Lorenzo Norsa, Maristella Pellegrino, Martina Fornaro, Valentina Fiorito, Marcello Lanari, Ester Giaquinto, Elvira Verduci, Maria Elisabetta Baldassarre, Antonella Diamanti

**Affiliations:** 1 Division of Clinical Nutrition, Regina Margherita Children’s Hospital, Città della Salute e della Scienza, 10126 Turin, Italy; igoreva@cittadellasalute.to.it; 2 Artificial Nutrition Unit Bambino Gesù, Children’s Hospital, IRCCS, 00165 Rome, Italy; teresa.capriati@opbg.net (T.C.); antonella.diamanti@opbg.net (A.D.); 3 Department of Transalational Medical Science, Section of Pediatrics, University of Naples Federico II, 80131 Naples, Italy; mispagnu@unina.it; 4 Department of Gastroenterology and Nutrition Unit, Meyer Children’s Hospital, 50139 Florence, Italy; laura.lacitignola@meyer.it; 5 Department of Pediatrics, “Burlo Garofolo” Hospital, University of Trieste, IRCCS, 34137 Trieste, Italy; grazia.dileo@burlo.trieste.it; 6 “Santobono-Pausillipon” Children’s Hospital, 80129 Naples, Italy; n.cecchi@santobonopausilipon.it; 7 Gastroenterology Unit, G. Gaslini Institute for Maternal and Child Health, IRCCS, 16145 Genova, Italy; paologandullia@gaslini.org; 8 Azienda USL-IRCCS di Reggio Emilia, Pediatrics Unit Arcispedale Santa Maria Nuova, 42123 Reggio Emilia, Italy; sergio.amarri@ausl.re.it; 9 Ped Gastroenterology and Nutrition Clinic, DIMEC, “Sant’Orsola” Hospital, 40138 Bologna, Italy; l.forchielli@tiscali.it; 10 Unit of Pediatrics, Department of Human Pathology in Adulthood and Childhood “G. Barresi”, University of Messina, 98122 Messina, Italy; dipasquale.valeria@libero.it; 11 Department of Pediatrics, Ospedale Sant’Anna, 22042 Como, Italy; barbaraparma79@hotmail.com; 12 Department of Pediatrics, AOU Ospedali Riuniti, Salesi’s Children Hospital, 60121 Ancona, Italy; simona.gatti@hotmail.it; 13 Local Health Care Service AUSL Romagna, 48121 Rimini, Italy; elisa.ravaioli@auslromagna.it; 14 Pediatric Department, “F. Del Ponte” Hospital, Insubria University, 21100 Varese, Italy; silvia.salvatore@uninsubria.it; 15 Department of Paediatrics, “Santa Maria delle Croci” Hospital, 48121 Ravenna, Italy; mmainetti111@gmail.com; 16 Paediatric, Hepatology, Gastroenterology and Transplantation, Hospital Papa Giovanni XXIII, 24127 Bergamo, Italy; lnorsa@asst-pg23.it; 17 “Cà Granda” Hospital, 20162 Milan, Italy; maristella.pellegrino@ospedaleniguarda.it; 18 Pediatrics Unit, “Morgagni-Pierantoni” Hospital, 47100 Forlì, Italy; martina.fornaro@auslromagna.it; 19 Pediatric Division of ”Santa Maria del Carmine” Hospital, Rovereto, 38068 Trento, Italy; valentina.fiorito@apss.tn.it; 20 Pediatric Emergency Unit of “Sant’Orsola” Hospital, 40138 Bologna, Italy; marcello.lanari@unibo.it; 21 Dietetic and Nutrition Center, Bufalini” Hospital, AUSL Romagna, 47521 Cesena, Italy; estergiaquinto@gmail.com; 22 “San Paolo” Hospital, 20142 Milan, Italy; elvira.verduci@unimi.it; 23 Neonatology and NICU Division, Policlinico “Aldo Moro” University Hospital, 70124 Bari, Italy; mariaelisabetta.baldassarre@uniba.it

**Keywords:** home enteral nutrition, home parenteral nutrition, oral nutritional supplements, children

## Abstract

Home Artificial Nutrition (HAN) is a safe and efficacious technique that insures children’s reintegration into the family, society and school. Epidemiological data on paediatric HAN in Italy are not available. Aim: to detect the prevalence and incidence of Home Parenteral Nutrition (HPN) and Home Enteral Nutrition (HEN), either via tube or mouth, in Italy in 2016. Materials and methods: a specific form was sent to all registered SIGENP members and investigators of local HAN centres, inviting them to provide the requested centre’s data and demographics, underlying diseases and HAN characteristics of the patients. Results: we recorded 3403 Italian patients on HAN aged 0 to 19 years from 22 centres: 2277 HEN, 950 Oral Nutritional Supplements (ONS) and 179 HPN programs. The prevalence of HEN (205 pts/million inhabitants) and HPN (16 pts/million inhabitants) has dramatically increased in Italy in the last 9 years. Neurodisabling conditions were the first indication for HEN by tube or mouth while HPN is mainly requested in digestive disorders. Conclusions: HAN is a widespread and rapidly growing treatment in Italy, as well as in other European countries. Awareness of its extent and characteristics helps improving HAN service and patients’ quality of life.

## 1. Introduction

Home Artificial Nutrition (HAN) provides extra hospital nutritional support in patients affected by malnutrition due to impairment of natural food intake, either absolute (children with neurodisabling conditions) or relative (children with organ failure requiring much more caloric intake than they autonomously can achieve or absorb) [1]. Home Enteral Nutrition (HEN) and Home Parenteral Nutrition (HPN), the two traditional ways of HAN providing, represent safe and effective therapeutic methods to obtain adequate nutritional status and the best possible quality of life [2]. However, the use of Oral Nutritional Supplement (ONS) for special medical purposes, regardless of the route of delivery, should be a further way of HAN providing, according to ESPEN and ESPGHAN indications [3]. HAN contributes towards insuring the child’s reintegration into family, society and school, and improves the child’s psychological condition [2]. Such complex and technical treatments should be provided by properly trained specialists in dedicated centres, including multidisciplinary nutrition support teams [2,4]. HAN impacts on the costs of health care because it allows reducing the number and length of hospitalizations [5]. Pediatric HAN programs in Italy seem to have increased in the last decade [6,7]. We have recently published a survey involving only patients with Intestinal Failure (IF) on Home Parenteral Nutrition (HPN) [8] and in 2013 the results of a survey on HEN that involved some Italian Administrative Regions [7]. Nevertheless, epidemiological data regarding management and magnitude of HAN in Italian children are not available. HAN support in children has certainly increased over the last years worldwide but data on its prevalence are very scarce. The existing reports outline fragmented and uncertain data on single type nutritional support (HEN or HPN) in adult and paediatric patients. The first report on exclusively paediatric data comes from the NEPAD registry of home and ambulatory enteral nutrition by the Spanish Society of Paediatric Gastroenterology, Hepatology and Nutrition [9]. An interesting phenomenon, reported by both European and American authors, is the underestimation of HAN activity recorded on scientific society registries compared to data gathered from home care companies, showing an approximately 90% shortfall in official data [10,11]. Knowing the epidemiology of HAN helps planning health-care funding and analysing the factors that can improve HAN service.

The aim of the survey was to assess prevalence and incidence of home enteral nutrition (HEN) by tube and mouth and home parenteral nutrition (HPN) in Italy and to define the main characteristics of the ongoing HAN programs.

## 2. Materials and Methods

An excel spreadsheet was sent to all registered SIGENP and SIP (Società Italiana di Pediatria—Italian Society of Pediatrics) members, inviting local investigators of HAN centres to participate without distinction of their paediatric specialization. Hospitals and Institutes involved in the survey are authorized to perform research and clinical studies by the Ministry of Health. Therefore, consent is acquired when patients are admitted to hospital, allowing their enrolment in non-active intervention clinical studies that guarantee anonymity. Thus, a separate ethics approval for the present survey was not required as no patients’ personal information was collected and the study design satisfied the criteria of an activity audit.

The following data have been included for all patients aged 0 to 19 years on HAN at the 31thDecember 2016:

- patient initials
- current age
- age at HAN beginning
- gender
- primary diagnosis requiring HAN
- number of HEN by tube and HPN programs started in 2016
- device for HEN delivery
- type of enteral feeds (by tube and mouth)
- type of parenteral bag (customized or standard)
- producer of parenteral bags (care company or local health service pharmacy)
- nursing service for HPN care (care company or local health service)

The data requested for every patient allowed us to exclude a double patients counting.

Primary endpoint of the survey was to assess the national paediatric HAN point prevalence at 31 December 2016 and the incidence in 2016 in Italy. Secondary endpoint was to assess the main characteristics of the different types of HAN.

Specifically, for home parenteral nutrition (HPN) we included a lower number of centres (16 vs. 19) but a higher number of patients followed (179 vs. 145), compared to our previous survey [12]. These discrepancies are due to the following considerations: (1) in the present survey we included patients affected not only by IF but also by extra-digestive diseases requiring HPN (e.g., oncologic patients without IF); (2) the previous survey included patients on HPN at 1 December 2016, while the present at 31 December 2016; in the meantime some centres stopped and other started HPN programs, therefore data from the two surveys did not exactly overlap in terms of contributors.

Statistical analysis: continuous variables were given as median and inter-quartile (IQ) range. Categorical variables were given as percentage of the entire group. HAN incidence and prevalence for 1,000,000 inhabitants from 0 to 19 years of age were assessed based on data from the National Institute of Statistics [8]. The latest estimate (January 2016) for population aged 0 to 19 years in Italy was overall 11,119,634 inhabitants, representing 18.3% of the whole population [8]. Statistical analysis was performed with STATA 11, StataCorp 4905 Lakeway Drive, College Station, TX 77845, USA and generation of figures were performed using Graph Pad 6 for Windows.

## 3. Results

Overall we recorded 3403 Italian patients on HAN aged 0 to 19 years.

**Figure 1:**
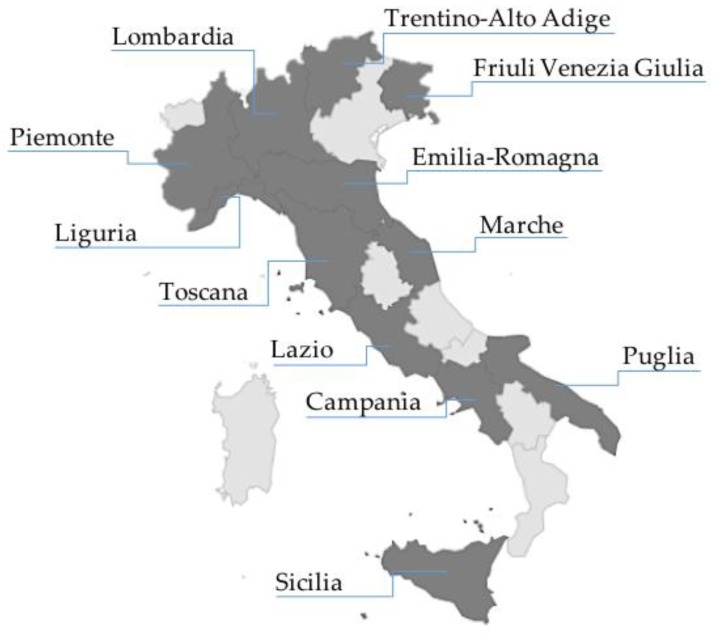
Legend: Geographical location and distribution of HAN centres on the Italian territory. Regions with HAN centres (dark grey); Regions without HAN centres (light grey).

In Table 1 we report the 22 centres that provided information regarding 12 out of the 20 Italian Administrative Regions and the type of HAN provided by each centre. Their geographical distribution and coverage of the Italian territory are reported in Figure 1. Some reference centres followed patients coming from more than one Region. Therefore, the available data gave a good representativeness of HAN activity all over the Italian territory.

In Table 2 are reported the demographic aspects of HEN by tube, HPN and HEN by mouth. Eight patients resulted on HPN combined with HEN by tube or mouth: they were affected by microvillus inclusion disease (1 patient); by pancreatic disease (1 patient); by graft versus host disease (2 patients) and by neurodisabling diseases (4 patients).

**Figure 2:**
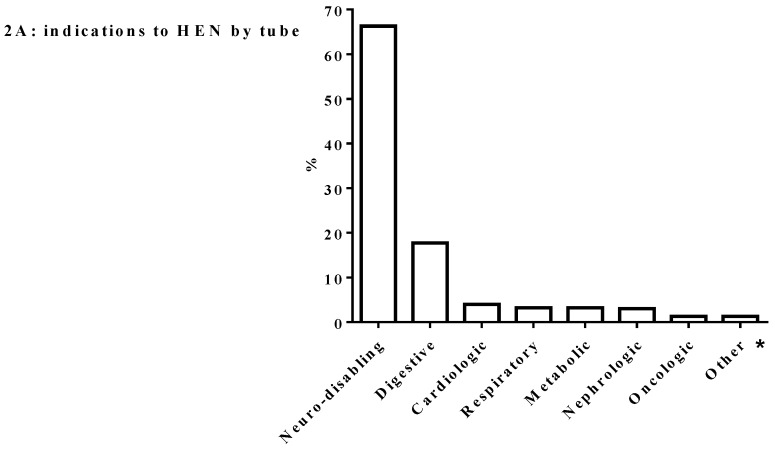

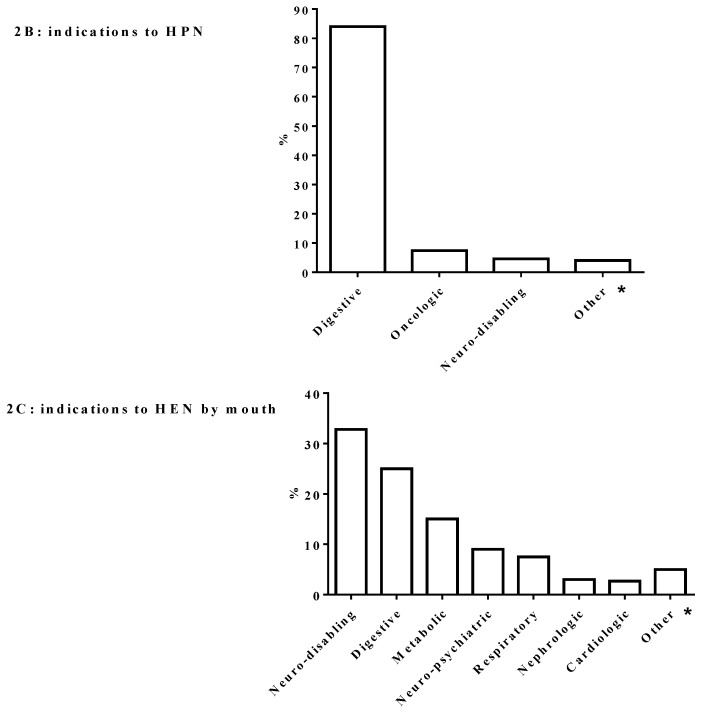
Indications to HAN. Legend: HAN: home artificial nutrition; HEN: Home Enteral Nutrition; HPN: Home Parenteral Nutrition; * Miscellaneous of: prematurity, failure to thrive, muscular dystrophy and post-acute malnutrition.

In Figure 2 we detail the primary diagnosis requiring HEN by tube and mouth and HPN. As reported, the neurodisabling diseases represented the most frequent indication to HEN while only a few patients (1%) with cancer required HEN support. A wide category of indications to HAN is represented by the digestive diseases that required HEN by mouth and tube and HPN. In particular, congenital or acquired oesophageal disorders indicated HEN by tube and mouth; inflammatory bowel disease required HEN by mouth while the three main categories of intestinal failure (IF) (short bowel syndrome, motility disorders and congenital enteropathies) accounted for more than 80% of HPN programs. Interestingly, in our series about 5% of HPN programs have been started in patients with neurodisabling conditions. About 9% of all indications to HEN by mouth were observed in patients with eating disorders, as part of the program of nutritional rehabilitation started in hospital. Some conditions not sufficiently frequent to be represented separately have been defined as “other”. In particular, for HEN programs, “other” included the following conditions: prematurity, failure to thrive, muscular dystrophy and post-acute malnutrition. In patients on HPN “other” included patients with inborn error of the metabolism, pancreatic diseases and immunodeficiency.

**Figure 3:**
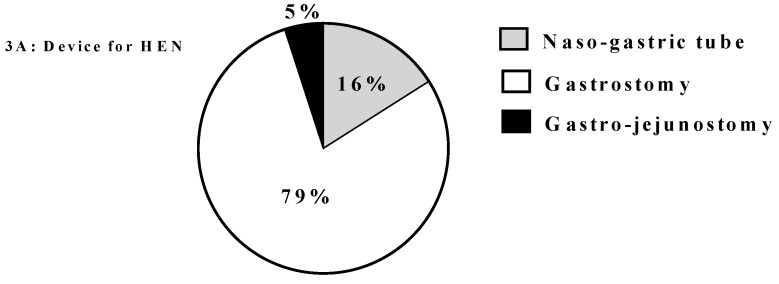

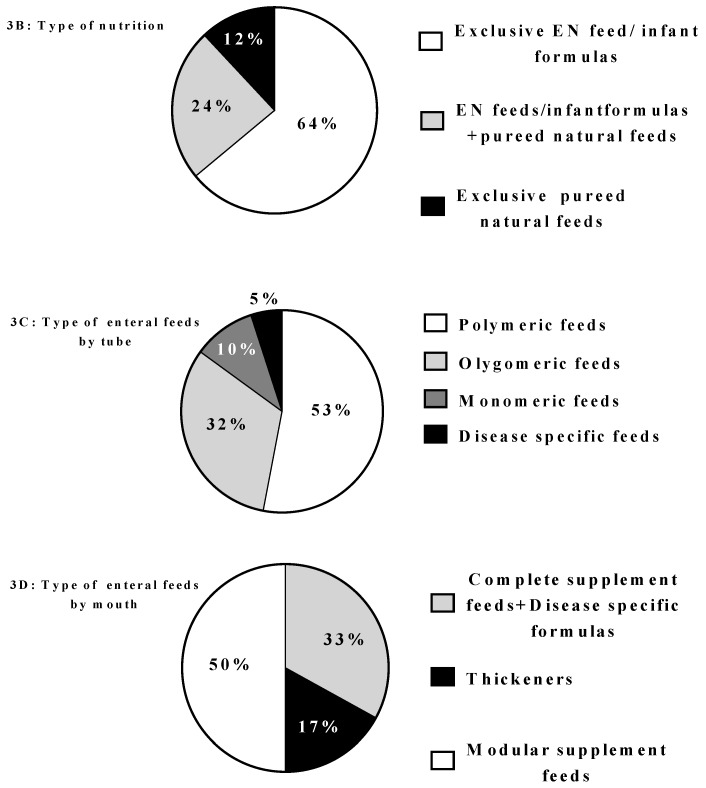
Characteristics of HEN. Legend: HEN: home enteral nutrition; EN: enteral nutrition.

**Figure 4:**
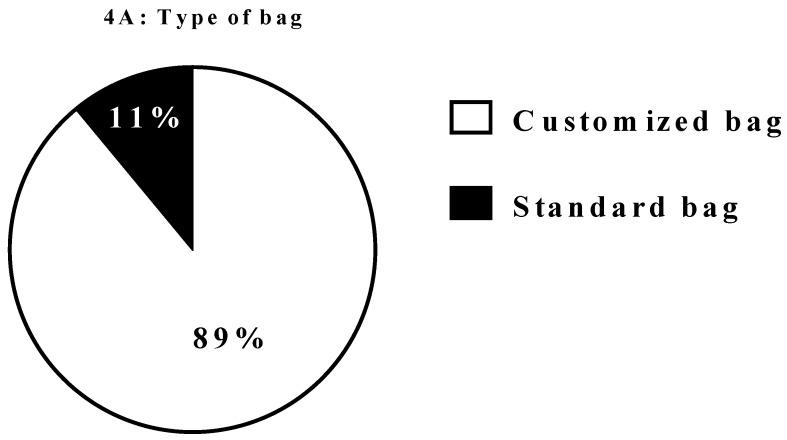

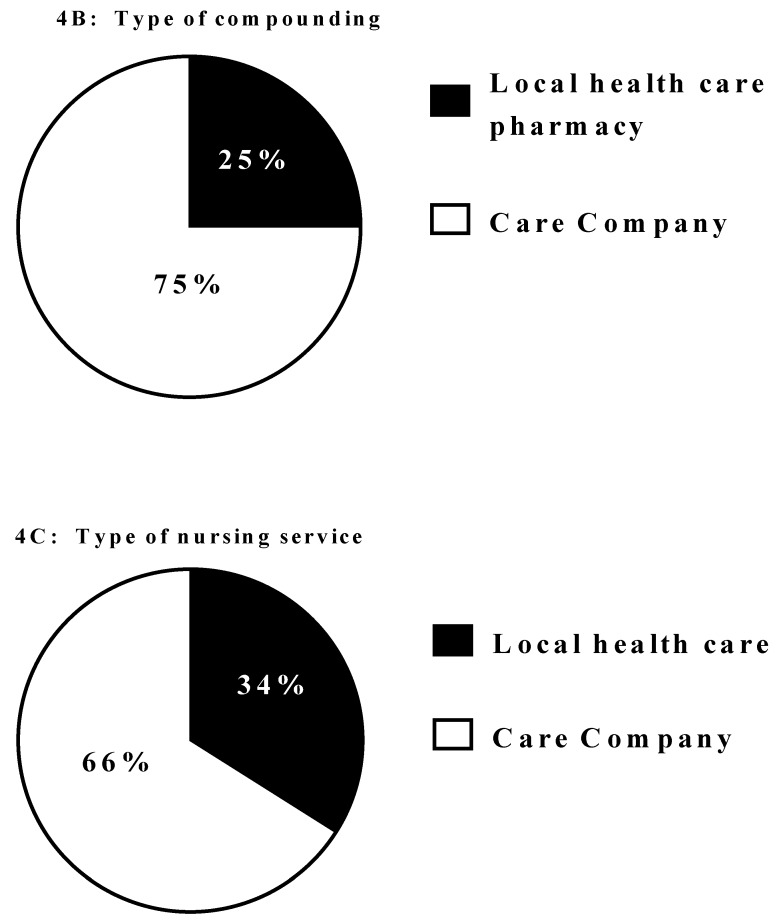
Characteristics of HPN. Legend: HPN. Home parenteral nutrition. Data on ONS provision were available in 17 out of 22 centres, 11 of which declared to have the entire furniture covered by regional funds whereas in 6 out of 17 centres the cost of ONS is only partially reimbursed.

Figure 3 and Figure 4 report the specific characteristics of HEN and HPN respectively. In several patients HEN by mouth was based on more than one type of ONS. In the present survey there were no patients who received HEN by naso-jejunal tube.

16 centres with overall 179 patients on HPN were detected by this survey. The characteristics of HPN programs have been previously described elsewhere [13]. The differences between HPN data gathered by the two surveys are reported in materials and methods.

## 4. Discussion

This survey shows a dramatic increase in HAN prevalence in paediatric patients in Italy as compared to previous surveys [6,14]. Interest in this type of nutritional support has expanded rapidly worldwide [15,16], supported by the progress in paediatric gastroenterology and nutrition techniques as well as all types of home-based care. Although few studies have been published, HAN is a safe and efficient technique that is compatible with a good quality of life and clinical outcomes, provided that the children and parents who attend the treatment receive adequate training and follow-up.

In our country, HEN and HPN are managed by specialist hospital centres. The existence of these teams and the availability of paediatric materials changed the approach to home nutritional care, that is no longer only reserved to patients with advanced diseases but can also be offered to all children who need nutritional support and can receive it at home. The prevalence of HAN in Italy detected by this survey was 306 patients per million of inhabitants <19 years and the incidence of HAN was 37.5 new paediatric patients per million of inhabitants <19 years through 2016. The previous surveys [6,9] registered an increasing rate of paediatric HAN patients in Italy +30% per year from 2005 to 2012 while our survey revealed an average increase of +36% per year. It cannot be excluded that a small number of paediatric patients may attend adult centres that did not adhere to this survey. We can also speculate that in the regions without proper HAN centres potential candidates to HAN may exist, which leads us to hypothesise a further increase of paediatric HAN prevalence should a better diffusion of specialist centres be possible in the foreseeable future. The last two issues may be the reason of the fuzzy outline of the prevalence figure described by the survey and this is one of the limitations of our study.

### 4.1. Home Enteral Nutrition (HEN)

HEN is the principal type of home artificial nutrition in Italian paediatric population. Including the ONS prescription, HEN represents 67% of all HAN programs. The prevalence of paediatric HEN has dramatically increased in European countries [15,16]. By the end of 2016 the prevalence of paediatric HEN in Italy was more than 6-fold that of 2005 [6]. Part of this growing evidence may be due to the specific paediatric focus of this survey, thus helping to detect more capillarily the real HEN distribution in our country. The previous Italian report gathered data (757 patients on HEN) only from four Italian Regional Reference Paediatric Centres [7]. HEN programs begin early in life, as showed in our previous report [7] and the starting age is lower than that reported by other authors [15]. The median duration of HEN significantly increased from 8.1 months (IQR 4.5–23.5) to 6 years (IQR0, 1–18.7) and this underlines the need for paediatric HAN centres to arrange transitional care of patients who survive through adolescence into adulthood. There are no guidelines or publications on transition of patients on enteral nutrition to adult healthcare services, while there are a few publications on HPN patients [17,13]. Transition of young patients suffering of chronic conditions, especially if congenital or infrequent in the adult services that need lifelong nutritional support, is one of the greatest challenges for health services and clinicians.

Regarding the primary diagnosis requiring HEN, by tube and mouth, this survey showed a low use of HEN in cancer patients. Data from the Spanish registry showed 15.3% of patients on HEN being affected by oncological diseases [9] versus 1% in our survey. Enteral nutrition is highly recommended in at-risk or malnourished cancer patients who are not able to cover their daily energy needs [18,19] providing, if necessary, partial parenteral support and reserving exclusive parenteral nutrition to those patients who fail to tolerate any enteral or oral supplementation [20].

### 4.2. Oral Enteral Nutrition (OEN)

The use of ONS, as part of HAN, is the newest contribution of our survey. ONS have a great potential in improving healthcare quality and lowering costs of healthcare in hospitalized paediatric patients [21]. The present survey showed a wide use of ONS in non-hospitalized children, able to eat orally, in order to treat protein-calorie malnutrition that accompanies many chronic conditions, often not fully addressed by paediatricians. To our knowledge, there are no papers on this topic. An increasing number of conditions may benefit from ONS to cover energy needs and prevent or treat malnutrition or provide specific nutrients. Some cases could require ONS for several years, as testified by the median duration of the supplementation in our survey of 1.3 years (IQR 0.3–6.8). This rises the problem of the reimbursement of ONS that varies by region and clinical condition and frequently has to be completely covered by the patient. Certainly, cost-effective studies testing the influence of ONS on health outcomes may help integrate ONS reimbursement in the actual regional policies, as it already happens for enteral nutrition programs. At present we are witnessing an increase in clinicians’ sensibility and industries’ interest on the matter, as shown by the ever growing assortment of paediatric ONS, albeit still poorer than that for adults.

### 4.3. Home Parenteral Nutrition (HPN)

This survey briefly reports data on HPN, given that prevalence, incidence, regional distribution and underlying diagnosis of HPN in paediatric chronic IF (CIF) patients in Italy have been recently reported elsewhere [12].

We detected some “new” contributors in the HPN population such as neurologic (4, 5%) and cancer patients (7, 3%) as well as the category of “other” (9%) mixed diagnosis. Managing intestinal failure in this patient group may be challenging. Specifically, neurodisabled patients, whose survival is progressively increasing, may need long term PN to cope with intestinal motility disorders [22]. We are facing some uncertainty about the possibility of returning these patients back to enteral feeding and ethical issues arise on the activation of HPN in these life-limiting conditions.

## 5. Strength and Limitations of the Study

This survey gave birth to the SIGENP Paediatric Home Artificial Nutrition Network, which collaborated to the implementation of the national registry of HAN, starting data gathering from 2017. It showed the prevalence of the treatment all over the Italian territory; this is the first survey reporting data exclusively from paediatric centres. The present survey has been inspired by evidence of the growing HAN activity in Italy, emphasized by the numerous scientific contributions of the Nutrition Area of SIGENP in national congresses and meetings over the last years. Having such specifics helped this survey to detect the most realistic picture of HAN activity in paediatric patients in Italy, thus allowing us to describe and discuss its extent and the new emerging features. It is a relevant issue that does help health care planning in order to improve HAN services and patients’ quality of life. The creation of the HAN Network will promote development of standards of care, exchanges between centres and patients’ access to HAN.

Nevertheless, we were not able to compare our findings with independently acquired data and estimate an eventual shortfall since HEN, HPN and especially HEN by mouth are not uniformly provided on the Italian territory and home care services are not common deliverers. Furthermore, paediatric patients followed by adult centres are not known as it has not been investigated. We couldn’t exclude the existence of potential candidates for HAN in regions without proper centres; however, the major HAN centres involved in the study followed patients from different regions.

## 6. Conclusions

This is the first survey that thoroughly describes paediatric HAN in Italy. Our data showed a steady increase of HEN and HPN and gave a first glance on the use of ONS in children affected by chronic diseases. The cost of HAN is significantly lower than the nutritional support in hospital and it allows to spare the adjunctive costs of malnutrition in these patients. The growing number of HAN programs, especially those activated in early life, and the complexity of the treatment underlines the necessity of a specialized management of home enteral and especially home parenteral nutrition. The treatment of specific conditions, like neurological and oncological diseases, have drastically improved patients’ life expectancy, raising the need for more attention in monitoring the nutritional needs and identifying the right timing for nutritional support by clinicians, in order to guarantee the cost-effectiveness of HAN and the patient’s quality of life.

## Figures and Tables

**Table 1:** Italian centres included in the survey.

Administrative Region	Centre	City	Type of HAN
Lombardia	“Papa Giovanni XXIII” Hospital	Bergamo	HEN, HPN
	“Sant’Anna” Hospital	Como	HEN
	“San Paolo” Hospital	Milano	HEN
	“F. Del Ponte” Hospital	Varese	HEN
	“Cà Granda” Hospital	Milano	HEN, HPN
Piemonte	“Regina Margherita” Hospital	Torino	HEN, HPN
Campania	University Hospital “Federico II”	Napoli	HEN, HPN
	“Santobono-Pausillipon” Hospital	Napoli	HEN, HPN
Puglia	University Hospital	Bari	HPN
Emilia-Romagna	“Sant’Orsola” Hospital	Bologna	HEN, HPN
	Local health care service	Rimini-Riccione	HEN, HPN
	“Bufalini” Hospital	Cesena	HEN
	“Morgagni-Pierantoni” Hospital	Forlì	HEN, HPN
	“Arcispedale Santa Maria” Hospital	Reggio Emilia	HEN, HPN
	“S. Maria delle Croci”Hospital	Ravenna	HEN, HPN
Sicilia	University General Hospital	Messina	HEN
Marche	“Salesi”Children’s Hospital	Ancona	HEN, HPN
Liguria	“Giannina Gaslini”Children’s Hospital	Genova	HEN, HPN
Lazio	“Bambino Gesù”Children’s Hospital	Roma	HEN, HPN
Trentino-Alto Adige	“Santa Maria del Carmine” Hospital	Rovereto	HEN
Friuli Venezia Giulia	“Burlo Garofolo”Children’s Hospital	Trieste	HEN, HPN
Toscana	“Meyer”Children’s Hospital	Firenze	HEN, HPN

HAN: home artificial nutrition; HEN: home enteral nutrition; HPN: home parenteral nutrition.

**Table 2:** Epidemiology of HAN in Italy.

	HEN by Tube	HPN	HEN by Mouth
Number of programs	2277	176	950
(% of overall HAN)	(70)	(5)	(25)
M/F (%)	43/57	45/55	51/49
Age at HAN beginning (years)	2.3	0.8	5.0
(median ± IQ range)	(0.3–9.7)	(0.1–1.8)	(1.6–12)
Current age (years)	7.2	4.8	9.7
(median ± IQ range)	(1.1–15.0)	(1.7–11.7)	(5.3–14.3)
Length of the program	6 years	3 years	1.3 years
(median ± IQ range)	(0.1–18.7)	(1.5–6.3)	(0.3–6.8)
Prevalence	204.7	15.8	85.5
(pts/million of inhabitants < 19 years)			
Incidence	35.9	1.6	/
(pts/million of inhabitants < 19 years)			

HAN: home artificial nutrition; HEN: home enteral nutrition; HPN. Home parenteral nutrition; IQ: interquartile.
